# Redescription and Molecular Characterisation of *Gnathia tridens* Menzies & Barnard, 1959 (Isopoda: Gnathiidae), a Presumed Ubiquitous Nearshore Isopod from the Temperate Northern Pacific

**DOI:** 10.1007/s11686-025-01005-2

**Published:** 2025-03-27

**Authors:** Anja Erasmus, Nico J. Smit, Claire A. Spitzer, Paul C. Sikkel, Niel L. Bruce, Kerry A. Hadfield

**Affiliations:** 1 Water Research Group, Unit for Environmental Sciences and Management, North-West University, Private Bag X6001, Potchefstroom, 2520 South Africa; 2 Department of Biology and Coastal and Marine Institute, San Diego State University, San Diego, USA; 3 Department of Marine Biology and Ecology, Rosenstiel School of Marine, Atmospheric, and Earth Science, University of Miami, Miami, FL 33124 USA; 4 Biodiversity and Geosciences Program, Queensland Museum, South Brisbane BC, PO Box 3300, Brisbane, QLD 4101 Australia

**Keywords:** Marine parasitic isopod, California, Systematic taxonomy, Temperate Northern Pacific Realm, Temporary fish parasite

## Abstract

**Purpose:**

*Gnathia tridens* Menzies & Barnard, 1959, is redescribed from material collected from San Diego, California and compared to the original description, as well as material held at the Natural History Museum of Los Angeles County and the Santa Barbara Museum of Natural History.

**Materials and Methods:**

A full redescription is given based on both morphological and molecular characteristics of the male using light and scanning electron microscopy, and COI mtDNA and ITS2 rDNA genes, respectively.

**Results:**

The key distinguishing characters that set *G. tridens* apart from other congeners are the equally trifid mediofrontal process, the mandible with a large incisor; the mesioventral margin anterior tip dorsally visible; pereonite 4 with distinct visible anterior constriction; and the three proximal tubercles on the antenna articles. Based on the molecular data for COI, the closest congener differs with 122 base pairs.

**Conclusion:**

Together, the combined morphological and molecular characterisation will provide a foundation for future, taxonomic, phylogenetic and biogeographical studies within the genus *Gnathia* and the Gnathiidae.

## Introduction

*Gnathia* Leach, 1814 [1] is the most species-rich genus within the family Gnathiidae with at least 141 known species [2]. According to Kim *et al*. [3], Ota *et al*. [4] and [5], 21 species within the genus *Gnathia* have previously been described and reported from the Temperate Northern Pacific (TNP) realm (see [6] for classification of various realms) (Table 1). Juvenile gnathiid isopods are well-known temporary fish parasites, while adults are free-living and can be identified by the distinct morphology of the adult male mandibles and frontal margin. Host records have been reported for some species, mostly those infesting chondrichthyans, while other species have been reported from specific habitats within brackish and marine waters. These include sandy and muddy substrata, as well as various forms of coral, algae, seagrass and kelp. Of the 21 species reported from the TNP, only *Gnathia trimaculata* Coetzee, Smit, Grutter & Davies, 2009 [7]  from Japan, has been molecularly characterised.

**Table 1:** Summary of currently known species of *Gnathia* Leach, 1814 from the Temperate Northern Pacific (TNP) [6]

Species	Total length (mm)	Type locality	Depth (m)	Habitat/Substratum Host (if known)
*Gnathia bungoensis* Nunomura, 1982 [16] (see also [17])	3.5*	Bansho River, Oita Prefecture, eastern coast of Kyusyu, southern Japan; 32° 58′ N, 131° 55′ E	2	Muddy colonies of green algae, *Ulva* spp.; near the sandy bottom of the estuary of Bansho-River; near the rocky shore, in colonies of brown algae, *Sargassum* spp.
*Gnathia capillata* Nunomura & Honma, 2004 [18]	7.6*	Sado Island, western Honshu, Japan	Unknown	Gill chamber wall Sting ray, *Dasyatis akajei*; several other chondrichthyan fishes
*Gnathia capitellum* Ota, Kohtsuka & Tanaka, 2021 [4]	2.1–2.5	Nabeta Bay, Izu Peninsula, Miura Peninsula, Japan; 34° 66′ 45.4″ N, 138° 94′ 12.4″ E	3–11	Dredging and muddy substratum *Platycephalus* sp., *Takifugu snyderi,*
*Gnathia clementensis* Schultz, 1966 [19]	8.5	San Clemente Canyon, off southern San Clemente Island, California, the United States; 32° 44′ 00″ N, 118° 12′ 45′ W	162	Grab sample containing manganese nodules
*Gnathia coronadoensis* Schultz, 1966 [19]	3.5	Coronado Canyon, off San Diego, California, the United States; 32° 30′ 42″ N, 117° 21′ 37″ W	182–812	Green mud and grey mud with H_2_S smell
*Gnathia derzhavini* Gurjanova, 1933 [20]	5.1	Peter the Great Bay, Sea of Japan, (south of Askold Island), Russia	121–110	Unknown
*Gnathia gurjanovae* Golovan, 2006 [21]	5	Peter the Great Bay, Sea of Japan, Russia	66	Clay-like silt, silted sand
*Gnathia hirsuta* Schultz, 1966 [19]	4	Santa Cruz Canyon, southern coast of Santa Cruz Island, California, the United States; 33° 56′ 03″ N, 119° 52′ 03″ W	218	Rocks and some green sand
*Gnathia koreana* Song & Ming, 2018 [22]	4.3–4.6	Yeogaekseon terminal, Geomuno Island, Yeosu-si, Jeollanam-do, South Korea; 34° 01′ 37″ N, 127° 18′ 27″ E	10	Organic-rich muddy sand
*Gnathia mutsuensis* Nunomura, 2004 [23] (see also [17])	2.1	Asamushi, Aomori Prefecture, northern Honshu, Japan	Unknown	Intertidal shore
*Gnathia nasuta* Nunomura, 1992 [24] (see also [17])	11.9–4.4	Off Tomioka, Reihoku-cho, Kumamoto Pref., Kyushu, western Japan; 32° 20′–22′ N, 130° 01′–03′ E	8.5–412	Sandy and muddy sediment
*Gnathia obtusispina* Kim, Kim & Yoon, 2023 [3]	3.2	Hongdo Island, Jeollanam-do (Province), southwestern South Korea; 34° 43′ 22.8″ N, 125° 11′ 59.5″ E	10	Rinsing bryozoans and macroalgae on the bedrock of sublittoral zones
*Gnathia productatridens* Menzies & Barnard, 1959 [8]	3.2	Off Summerland, Santa Barbara, California, the United States; 34° 14′ 50″ N, 119° 32′ 25″ W	94	Green silt
*Gnathia rectifrons* Gurjanova, 1933 [20]	6	Peter the Great Bay, Sea of Japan, Russia	80–88	Unknown
*Gnathia sanrikuensis* Nunomura, 1998 [25] (see also [17])	2.8–3	Otsuchi Bay, Iwate Prefecture, northern Japan; 39° 20′–21′ N, 141° 53′–58′ E	42	Sandy sediment
*Gnathia schmidti* Gurjanova, 1933 [20]	5.5	Vladimir Bay, Sea of Japan, Russia; 43° 56′ N, 135° 56′ E	8	Unknown
*Gnathia steveni* Menzies, 1962 [26]	2.3	Bahia de San Quintin, Baha California Mexico, c 30° 26′ 34.9″ N, 15° 57′ 27.2″ W	Unknown	Intertidal rocks
*Gnathia tridens* Menzies & Barnard, 1959 [8]	3	Point Conception, San Diego, California, the United States; 34° 26′ 54″ N, 120° 28′ 17″ W	11	Kelp habitat; red algae
*Gnathia trilobata* Schultz, 1966 [19]	5	Coronado Canyon, off San Diego, California, the United States; 32° 30′ 42″ N, 117° 21′ 37″ W	812–976	Green sand and mud
*Gnathia trimaculata* Coetzee, Smit, Grutter & Davies, 2009 [7] (see also [5])	4–5.4	Off Lizard Island, Australia; 14° 40′ 54.68″ S, 145° 26′ 53.72″ E	Unknown	Rocky shores First and second stage larvae: external surface of *Enneapterygius etheostomus*, *E. miyakensis*, and *Springerichthys bapturus*; Third stage larvae: gill filaments of *Carcharinus melanopterus* and* C. amblyrhynchos*
*Gnathia tuberculata* Richardson, 1909 [27]	3.5*	Off Sudzii Misaki Light, east of Noto Peninsula, Japan; 37° 22′ 30″ N, 137° 47′ 00″ E	1100	Green mud and grey mud with H_2_S smell

*Total length including mandibles, excluding antennae

Where available, additional descriptors such as the substratum where free-living adults have been collected, and hosts where parasitic larval stages were collected, are included (adapted from [3])

*Gnathia tridens* Menzies & Barnard, 1959 [8], was described more than 65 years ago from specimens collected off Point Conception, southern California (USA) within the TNP. In 1997, Wetzer and Brusca [9] provided a short description of *G. tridens,* as well as an illustration of the habitus which they labelled as the male holotype and a female paratype and a key to the Californian species of *Gnathia*. A considerable amount of interesting and valuable information on *G. tridens* was also captured in the newsletters of the Southern California Association of Marine Invertebrate Taxonomists (SCAMIT) [10], however, what they considered to be the male of *G. tridens* does not correspond to some critical aspects of the original description [8] or the notes provided by Wetzer and Brusca [9], specifically characters such as the cephalon (herein referred to as cephalosome) lacking setae and tuberculation. The most recent mention of *G. tridens* was in a review and guide to the isopods of the Southern California Bight by Stebbins and Wetzer [11], which included a key to Gnathiidae. In this review [11], the distribution of *G. tridens* extends from southern California northwards to British Columbia (Canada), however, it is not clear how the specimens from Canada (and elsewhere) were identified [12–15]. Although the species appears to be widespread, this cannot be confirmed as little or no taxonomic evidence was provided, and in several cases no specimens were retained. There is also no available data that corroborates an extended distribution beyond southern California for *G. tridens*. Currently, available data lack sufficient information on the key morphological characters needed for the consolidation of this species. Therefore, a comprehensive taxonomic redescription, integrated with molecular data, is essential. The molecular characterisation will also enable researchers to identify *G. tridens* males and distinguish this species from other, possibly undescribed, species as well to unambiguously identify females and parasitic larval stages of *G. tridens* when collected in the absence of males.

## Materials and Methods

### Field Methods

**Fig. 1:**
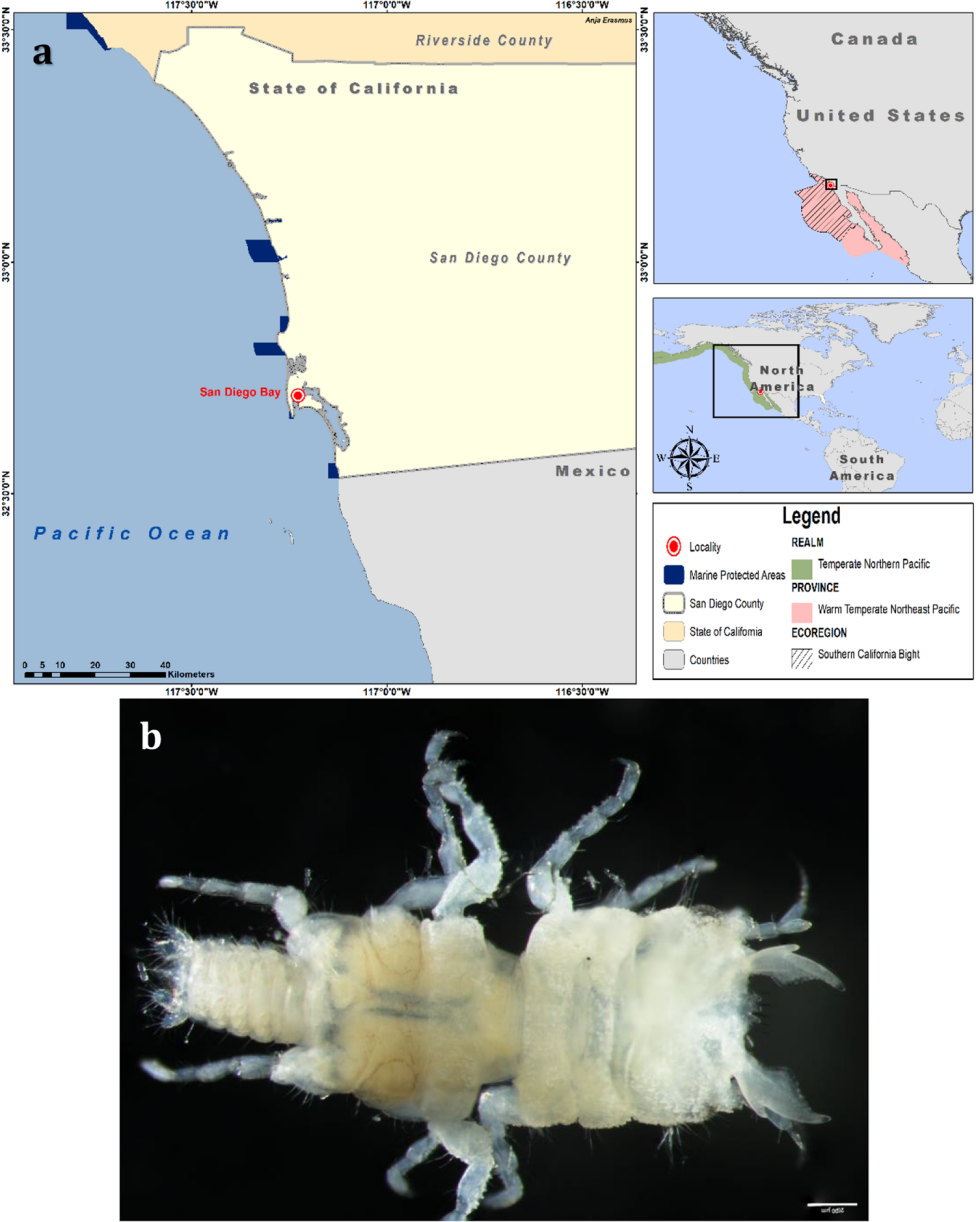
**a** Study area map of San Diego Bay, California, indicating the realm, province and ecoregion within the Marine Ecoregions of the World (MEOW); **b** *Gnathia tridens* male voucher material (LACM 36831). Scale bar: 200 µm. Marine region spatial data was obtained via ArcGIS Hub based on the data from [6]

Specimens were collected from April 2016 through April 2018 at depths of 10–12 m as described by [28] during a trapping study off San Diego, California, USA (32° 42′ 44″ N, 117° 13′ 37″ W) (Fig. 1). Host fish were collected under permits issued by the California Department of Fish and Wildlife (SC-13350) and handled according to the approved SDSU IACUC protocol (APF 15-10-013A). Adult males were moulted from third-stage pranizae in the laboratory following [29], and preserved in varying grades of ethanol for morphological study and molecular sequencing, respectively. In addition, photographs of the type material (previously AHF Type No. 5711) deposited at the Natural History Museum of Los Angeles County [8], as well as photographs of *Gnathia tridens* specimens (SBMNH 669829; SBMNH 694194; SBMNH 697320; SBMNH 697452) collected during benthic trawling surveys in Santa Barbara County, California from 1982 to 1985 [9] provided by the Santa Barbara Museum of Natural History, were obtained for comparison.

### Identification Methods

The research conducted at North-West University was granted ethical clearance under the reference number NWU-00784-24-A5. Preserved specimens were cleaned and allocated for various identification techniques including light and scanning electron microscopy and gene sequencing. Morphological characterisation was done using lignin pink-stained specimens (whole and dissected) as described for light and scanning electron microscopy in [30]. This same protocol was followed for the illustrations. DELTA descriptions were made with terminology based on [31] and [32]. The total length of the habitus was measured from the frontal margin (including the processes and excluding the mandibles), mid-dorsally, to the midpoint of the pleotelson [32]. Voucher material is deposited in the Natural History Museum of Los Angeles County (LACM).

### Molecular Characterisation

Mitochondrial DNA was isolated from two male specimens using the manufacturer’s protocol for animal tissue extraction of the NucleoSpin^®^ Tissue Genomic DNA Tissue Kit (Macherey–Nagel, Düren, Germany). Amplification of COI mtDNA and ITS2 rDNA genes was done using specific polymerase chain reaction (PCR) protocols and primers (LCO1490 and HCO2198, and 3S-forward and ITS2.2-reverse, respectively) (Table 2). Polymerase chain reaction (PCR) protocols, reactions and conditions were followed as in [26]. Thereafter, sequences were trimmed, assembled and edited using bioinformatic software, Geneious R7.1.3 (RRID: SCR_010519) [33]. Sequences were compared to known sequences of *Gnathia* available on GenBank and BOLD and subsequently submitted to GenBank (COI: PV213449; PV213450 and ITS2: PV211460 and PV211461).

**Table 2:** Gene regions of COI mtDNA and ITS2 rDNA, with selected PCR primers, used to amplify DNA for molecular characterisation of *Gnathia tridens*

Gene region	Primers	Nucleotide sequence	References
COI mtDNA	LCO1490	5′-GGTCAACAAATCATAAAGATATTGG-3′	Folmer *et al*. (1994) [34] Shodipo *et al*. (2021) [35]
	HCO2198	5′-TAAACTTCAGGGTGACCAAAAAATCA-3′	
ITS2 rDNA	3S-Forward	5′-GGTACCGGTGGATCACGTGGCTAGTG-3′	Grutter *et al*. (2000) [36]
	ITS2.2-Reverse	5′-CCTGGTTAGTTTCTTTTCCTCCGC-3′	

*AHF* Allan Hancock Foundation; *DELTA* DEscriptive Language for TAxonomy; *LACM* Natural History Museum of Los Angeles County; *MEOW* Marine Ecoregions of the World; *NWU-WRG* North-West University Water Research Group; *SBMNH* Santa Barbara Museum of Natural History; *SEM* scanning electron microscopy; *TNP* Temperate Northern Pacific

## Results

### Taxonomy

Suborder Cymothoida Wägele, 1989

Superfamily Cymothooidea Leach, 1814

Family Gnathiidae Leach, 1814

*Gnathia* Leach, 1814

Type species. *Gnathia termitoides* Leach, 1814 (= *Gnathia maxillaris* (Montagu, 1804) [37]); type by original designation [31].

*Gnathia tridens* Menzies & Barnard, 1959

*Gnathia tridens* Menzies and Barnard, 1959 [8]: 29, fig. 23.—Schultz, 1969 [38]: 229, fig. 365.—Wetzer *et al*. 1991 [39]: 36, 46.—Wetzer and Brusca, 1997 [9]: 47–49, figs 1.19, 1.20.—Spitzer *et al*. 2022 [28]: 69, fig. 2 photo.—Stebbins and Wetzer 2023 [11]: 5, 27, 29, 89–90, 164, fig. 12G.

Not *Gnathia tridens*.—Lissner *et al*. 1986 [12]: 29.—McLaughlin *et al*. 2005 [13]: 194.—Espinosa-Pérez *et al*. 2009 [14]: 229, table 1.—Macdonald *et al*. 2010 [15]: 20, table 2. [all = *Gnathia* sp.].

Excluded.—SCAMIT reports by [10] and [40]; Outer Continental Shelf (OCS) study by [41].

**Material examined.** 4 ♂ voucher specimens (2.4–3.1 mm TL) LACM 36831; LACM 36832, collected and reared as stated in [24], off San Diego, California, USA, 32° 42′ 44″ N, 117° 13′ 37″ W (LACM 36831; LACM 36832).

**Other material:** 3 ♂♂ (2.3–3.1 mm TL) dissected and stained with lignin pink used for light microscopy, same information as voucher. 1 ♂ (2.4 mm TL) prepared and used for SEM, same information as voucher. 2 ♂♂ (2.3–2.6 mm TL) used for genetic characterisation of COI mtDNA and ITS2 rDNA genes (GenBank numbers COI: PV213449; PV213450 and ITS2: PV211460 and PV211461), same information as voucher. 3 ♂♂ (2.3–4.1 mm TL) examined for variation in frontal margin, same information as voucher (in the collection of the NWU-WRG). Photos of the type material provided by LACM were examined for direct comparison.

**Type locality.** Point Conception, 34° 26′ 54″ N, 120° 28′ 17″ W, California [8].

**Distribution.** Confirmed records are Point Conception [8] and San Diego [28 and present material], in California, USA.

**Habitat.** Bottom of dead kelp fragments and red algae [8], rocky reef, corals, seagrass, sediment, and kelp forest [28] at depths of 10–12 m.

**Host.** *Heterostichus rostratus* (Giant kelpfish) [28].

**Fig. 2:**
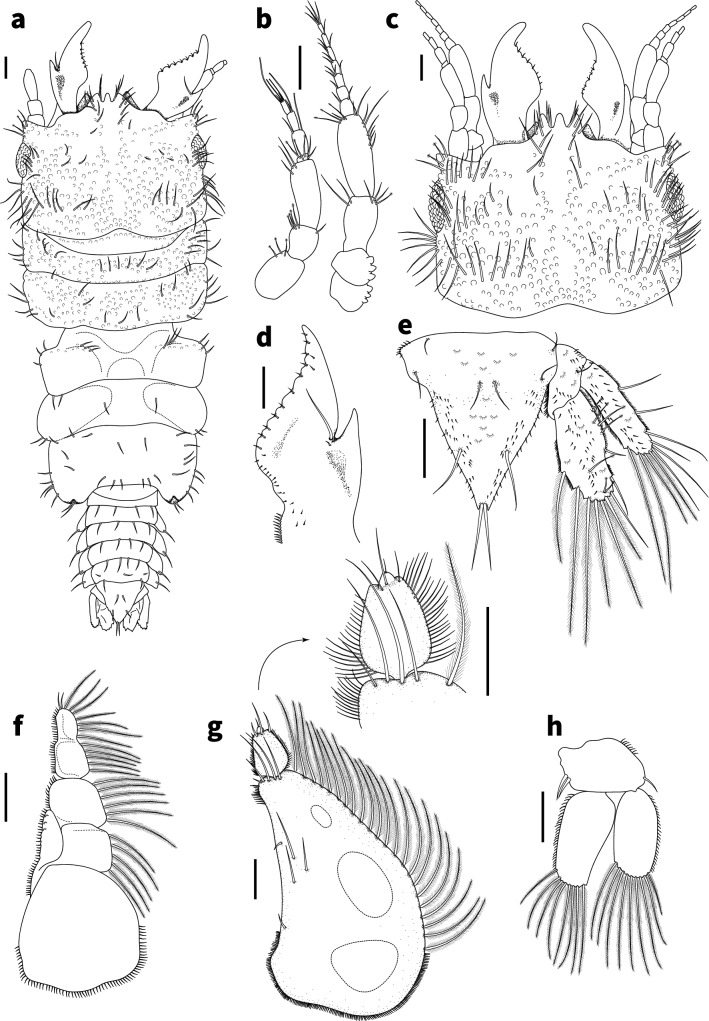
*Gnathia tridens,* male (2.6 mm TL) **a** habitus dorsal view (LACM 36831) **b** antennae **c** dorsal view of cephalosome with frontal margin and mandibles **d** right mandible dorsal view **e** pleotelson and uropod **f** maxilliped **g** pylopod with detail of articles 2 and 3 **h** pleopod 2. Scale bars: 100 µm

**Fig. 3:**
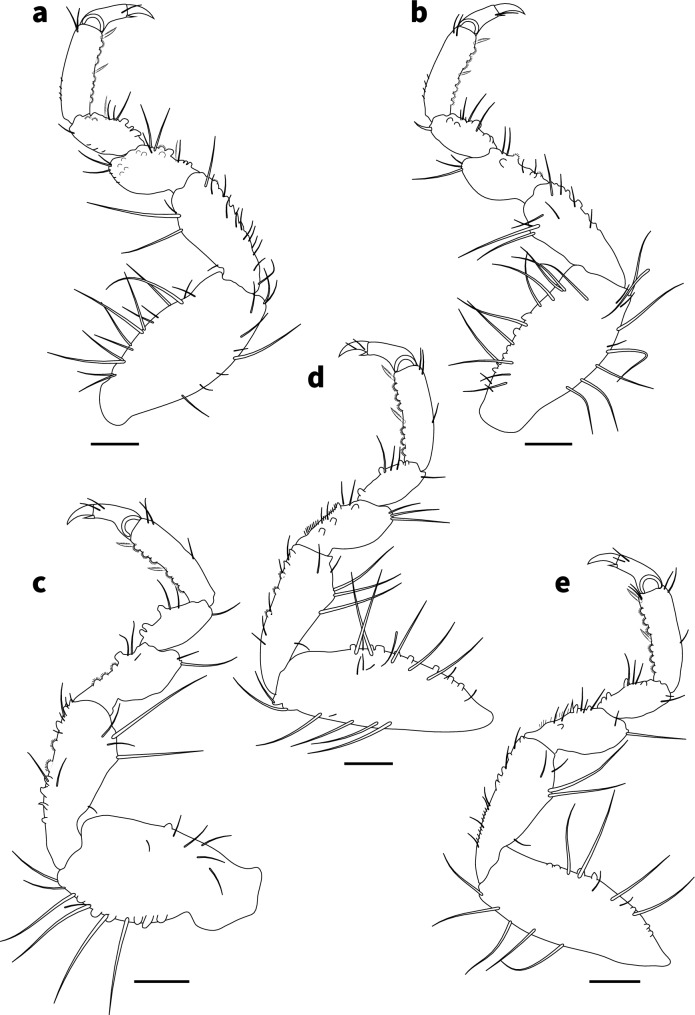
*Gnathia tridens*, male (3.1 mm TL) **a**–**e** right pereopods 2–6, respectively. Scale bar: 100 μm

**Fig. 4:**
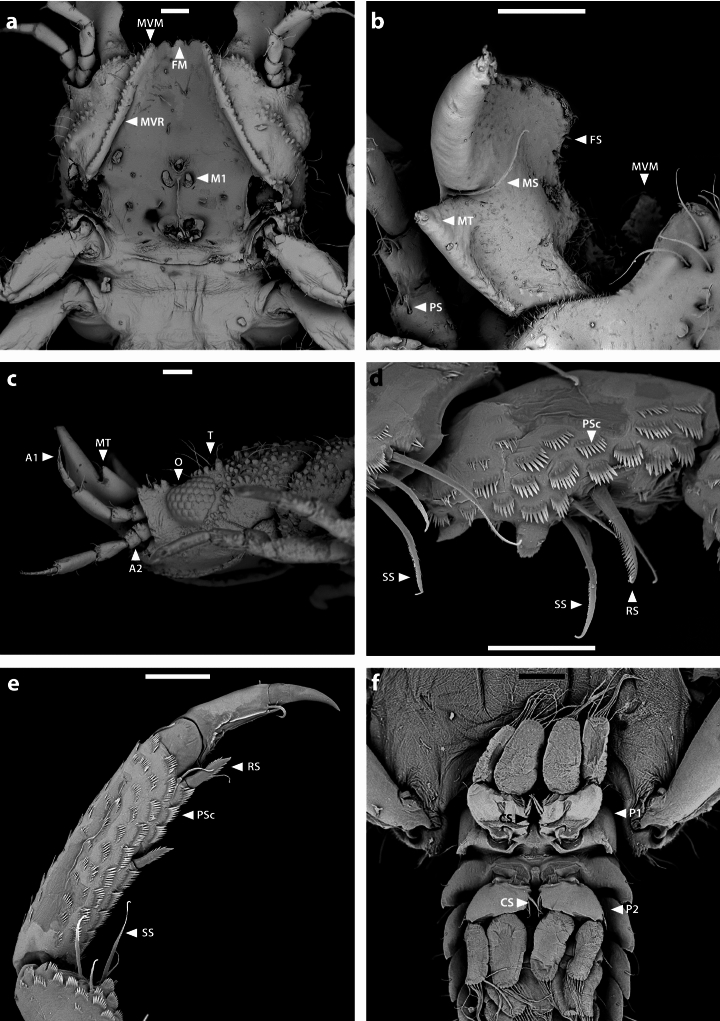
*Gnathia tridens*, male scanning electron microscope images. Cephalosome (**a**) ventral (**b**) dorsal (**c**) lateral, with three proximal tubercles on antenna peduncle article 1 and 2 **d** various types of setae on carpus of pereopod **e** ventral view of pereopod from propodus to unguis **f** ventral view of pleopod 1 and pleopod 2 with no appendix masculina. Scale bars: 100 μm (**a**–**c**, **f**); 50 μm (**d**, **e**). *A2* antenna; *A1* antennula; *CS* coupling seta/e; *O* eye ommatidia; *FS* fringe seta/e; *FM* frontal margin; *MS* mandibular seta/e; *MT* mandibular tooth; *M1* maxilla 1; *MVM* mesioventral margin; *MVR* mesioventral ridge; *PSc* pectinate scale/s; *P1* pleopod 1; *P2* pleopod 2; *RS* robust seta/e; *SS* serrate seta/e; *T* tubercles

***Description of adult male*** (Figs. 2, 3, 4):

*Body* (Figs. 1b, 2a) 2.1 times as long as greatest width, widest at pereonite 2; dorsal surfaces with tubercules anteriorly and smooth posteriorly, sparsely setose, chromatophores not apparent in fixed specimens. *Cephalosome* (Figs. 2c, 4c) rectangular, 0.6 as long as wide, lateral margins parallel, posterior margin medially concave; dorsal surface with numerous granules and tubercles around eyes; *dorsal sulcus* wide, shallow, short; translucent region absent; *paraocular ornamentation* weakly developed and with several tubercles and setae, posterior median tubercle present; with lateral tubercles. *Frontal margin* (Figs. 2a, c, 4a) with processes. *External scissura* present, wide, shallow. *Mediofrontal process* present, strong, produced, equally trifid, with 3 simple setae on either side. *Superior frontolateral process* absent. *Inferior frontolateral process* absent. *Mesioventral margin* straight; granular and setose; anterior tip dorsally visible. *Supraocular lobe* pronounced, pointed, accessory supraocular lobe not pronounced. *Eyes* elongate, 0.3 as long as cephalosome length, contiguous with head surface, ommatidia arranged in rows, eye colour not apparent in fixed specimens.

*Pereon* (Figs. 1b, 2a) lateral margins sub-parallel, with few setae; anteriorly with numerous fine granules. *Pereonite 1* not fused dorsally with cephalosome; dorsolateral margins fully obscured by cephalosome. *Pereonites 2 and 3* wider than pereonite 1. *Pereonite 4* with anterior constriction, median groove present. *Areae laterales* present on pereonite 5; dorsal sulcus wide. *Pereonite 6* with weak lobi laterales; lobuii weak, notched. *Pereonite 7* 4 times longer than wide. *Pleon* epimera dorsally visible on all pleonites. *Pleonite* lateral margins with 1 pair of simple setae, with 2 pairs of simple setae medially.

*Pleotelson* (Figs. 1b, 2a, e, 4f) 1.1 times as long as anterior width, partially covered in pectinate scales and covered in fringe setae; lateral margins finely serrate, anterolateral margins weakly concave, with 2 pairs of submarginal setae; posterolateral margin distally weakly concave, with 1 pair of submarginal setae; mid-dorsal surface with 1 pair of sub-median setae, apex with 2 setae.

*Antennula* (Figs. 2b, 4c) 0.7 times shorter than antenna; article 2 0.6 times as long as article 1; article 3 1.8 times as long as article 2, 2.25 times as long as wide; flagellum 1.1 times as long as article 3, with 4 articles; article 1 with 3 penicillate setae and 2 simple setae; article 2 with 1 aesthetasc seta and 1 simple seta; article 3 with 1 aesthetasc seta and 1 simple seta; article 4 with 3 aesthetasc seta and 2 simple setae. *Antenna* (Figs. 2b, 4c) peduncle with 4 articles; article 1 and 2 each with three distinct proximal tubercles; article 3 1.5 times as long as wide, 1.5 times as long as article 2, with 1 penicillate seta, and 5 simple setae; article 4 1.6 times as long as article 3, 2.4 times as long as wide, with 3 penicillate setae, and with 9 simple setae; flagellum 1.2 times as long as article 4, flagellum 1.9 times as long as article 3, with 7 articles, terminating with 4 simple setae.

*Mandibl*e (Figs. 2d, 4b) 0.7 as long as wide, and as long as length of cephalosome, triangular, weakly curved; apex 21% of total length; mandibular seta present. *Carina* present, smooth along proximal half. *Incisor* elevated, standing clear of surface, distal denticulation absent. *Blade* present, dentate, unevenly convex, midventrally convex, dentate along 60% of margin, bearing 6–7 small teeth. *Pseudoblade* absent. *Internal lobe* absent. *Dorsal lobe* absent. *Basal neck* short. *Erisma* present. *Lamina dentata* absent.

*Maxilliped* (Fig. 2f) 5-articled; article 1 lateral margin with continuous marginal scale-setae; article 2 lateral margin with 5 plumose setae; article 3 lateral margin with 6 plumose setae; article 4 lateral margin with 5 plumose setae; article 5 lateral margin with 7 plumose setae, and 2 simple setae; endite extending to mid-margin of article 3; with no coupling setae.

*Pylopod* (Fig. 2g) article 1 1.6 times as long as wide; with 3 distinct areolae; without distolateral lobe; posterior and lateral margins forming rounded curve; lateral margin with 22–26 large plumose setae; mesial margin with scale-setae on distal part only; surface 5–6 simple setae present; distal margin with 5–6 simple setae; article 2 1.4 times as long as wide; with 3 simple setae; article 3 min and partially fused to article 2, with 1 seta.

*Pereopods 2–6* (Fig. 3a–e) with long simple setae, and pectinate scales unevenly distributed along the inner margin of propodus, carpus, merus, and ischium; propodus distal robust setae (RS) slightly longer than proximal RS; inferior margins with weak tubercles, pereopod 2 with tubercles on ischium to carpus; pereopod 2 basis 2.3 times as long as greatest width, superior margin with 14 setae, inferior margin with 9 setae; ischium 1.5 times as long as basis, 2.3 as long as wide, superior margin with 4 setae, inferior margin with 12 setae; merus 0.5 times as long as ischium, 1.2 times as long as wide, superior margin with 3 setae; superior margin with bulbous protrusion; inferior margin with 5 setae; carpus 0.5 times as long as ischium, 1.7 times as long as wide, superior margin with 1 seta, inferior margin with 4 setae; propodus 0.7 times as long as ischium, 3.1 times as long as wide, superior margin with 2 simple setae, superior margin with 1 penicillate seta, inferior margin with 1 simple seta, no short setae, and 2 RS; dactylus 0.4 times as long as propodus; pereopods 3 and 4 similar to pereopod 2; pereopod 5 similar to pereopod 6; pereopod 6 with tubercles on basis to carpus; basis 2.7 times as long as greatest width, superior margin with 5 simple setae, and 2 penicillate setae, inferior margin with 5 setae; ischium 0.7 times as long as basis, 2.4 times as long as greatest width, superior margin with 5 setae, inferior margin with 5 setae; merus 0.6 times as long as ischium, 1.8 times as long as wide, superior margin with 4 setae, inferior margin with 6 setae, with dense patch of scale-setae; carpus 2 times as long as ischium, 1.3 times as long as wide, superior margin with 2 setae, inferior margin with 3 setae; propodus 1.3 times as long as ischium, 2.8 times as long as wide, superior margin with 3 setae, inferior margin with 2 simple setae, and 2 RS; dactylus 2.2 times as long as propodus.

*Penes* composed of non-prominent openings, almost flush with ventral surface of pereonite 6, penial process as long as basal width.

*Pleopod 2* (Figs. 2h, 4f) *exopod* 2.3 as long as wide, distally broadly rounded, with 9 plumose setae; endopod 1.8 times as long as wide, distally narrowly rounded, with 8 plumose setae; appendix masculina absent; peduncle 1.5 times as wide as long, mesial margin with 2 coupling setae, lateral margin with 1 simple seta.

*Uropod* (Fig. 2e) rami extending to pleotelson apex, apices broadly rounded. *Peduncle* with no dorsal setae. *Uropod endopod* 1.7 times as long as greatest width, dorsally with 7 penicillate setae; lateral margin straight, with 3 simple setae; proximomesial margin weakly convex, with 6 long plumose setae. *Uropod exopod* not extending to end of endopod, 3.3 times as long as greatest width; lateral margin weakly sinuate, with 6 simple setae; proximomesial margin straight, with 4 long plumose setae.

### Remarks

*Gnathia tridens* can be identified by the equally trifid processes on the mediofrontal process (with all three acute) that are longer than wide, the mandible with a large incisor (mandibular tooth), the mesioventral margin anterior tip dorsally visible, pereonite 4 with distinct visible anterior constriction separating pereonite 4 from pereonite 3, and the antenna peduncle articles 1 and 2 each with three proximal tubercles.

The material examined here agrees with the original description of *G. tridens* [8] based on the body size, trifid frons (herein referred to as frontal medial margin processes), third segment narrowed, inner face of the mandibles bearing 6–7 small teeth and the outer face bearing a large tooth (herein referred to as incisor). Additional distinguishing features not provided in the original description and subsequent notes on this species include: mesioventral margin anterior tip dorsally visible and antenna (A2) peduncle articles 1 and 2 each with three proximal tubercles.

*Gnathia tridens* may be identified and separated from all congeners within the TNP by the equally trifid frontal margin. In addition, *Gnathia magdalenensis* Müller, 1988 [42] from northern Colombia, *Gnathia trilobata* Schultz, 1966 [19] from the Coronado Canyon, *Gnathia vellosa* Müller, 1988 [42] from northern Colombia, and *Gnathia virginalis* Monod 1926 [43] from the Virgin Islands, may be distinguished by variations in their trifid frontal margin, none of which are equal in size. *Gnathia magdalenensis* can be distinguished by the subequal mediofrontal process and inner lobe on the mandible, while in *G. trilobata* the frontal margin is strongly produced with three mediofrontal processes. *Gnathia vellosa* appears closely related to *G. virginalis,* with both having granular body surfaces and three processes on the frontal margin. However, they differ in body size, and there is a distally notched carina on the mandible in *G. vellosa*, and a rounded mandibular carina in *G. virginalis. Gnathia tridens* can be further distinguished from *G. virginalis* by the presence of a mandibular seta, a 3-articled pylopod, and having 4 and 7 plumose marginal setae on the uropodal exopod and endopod, respectively.

In the original description by Menzies and Barnard [8], the authors mention that *G. tridens* is similar to *Gnathia africana* Barnard, 1914 [44], an intertidal species from the south and west coasts of South Africa. *Gnathia africana* was redescribed in 1999 [45], providing further morphological characters that clearly distinguish it from *G. tridens*. The two species can be distinguished by the produced equally trifid frontal margin present in *G. tridens,* the presence of frontolateral processes and inferior mediofrontal process that is divided in two in *G. africana*, supraocular ornamentation prominent in *G. africana* absent in *G. tridens*, and the three proximal tubercles of the antenna in *G. tridens* absent in *G. africana*.

### Molecular Phylogeny

Two consensus sequences of *Gnathia tridens* males were successfully acquired for COI mtDNA and ITS2 rDNA genes, respectively. These are the first sequences obtained for *Gnathia tridens*, and for any *Gnathia* species from the TNP. The sequences ranged from 623–640 base pairs (bp) within a 0.2%–0.4% divergence for COI (see Table 3) and ITS2, respectively. Due to limited published COI sequences available for species of *Gnathia* on both GenBank and BOLD, with only one known species, *Gnathia jimmybuffetti* Erasmus *et al*. [30], with comparable sequences for ITS2 rDNA, phylogenetic trees were not constructed.

**Table 3:** Genetic matrix for newly obtained COI sequences of *Gnathia tridens*, indicating the percentage similarity below the diagonal division and the nucleotide p-distances above the division

COI sequences for *Gnathia* spp.		1	2	3	4	5	6	7	8	9	10	11
**1**	**PV213449** ***Gnathia tridens***		**0.000**	0.318	0.264	0.322	0.335	0.287	0.282	0.328	0.284	0.327
**2**	**PV213450** ***Gnathia tridens***	**100**		0.319	0.264	0.321	0.338	0.288	0.284	0.327	0.284	0.329
3	MW804340 *Gnathia camuripenis*	68.2	68.1		0.313	0.318	0.313	0.305	0.325	0.320	0.272	0.313
4	OR064533 *Gnathia jimmybuffetti*	73.6	73.6	68.7		0.307	0.342	0.301	0.270	0.330	0.292	0.332
5	AB713956 *Gnathia limicola*	67.8	67.9	68.2	69.3		0.313	0.322	0.337	0.387	0.308	0.333
6	AB713961 *Gnathia maculosa*	66.2	65.9	68.3	65.5	68.3		0.326	0.345	0.363	0.303	0.260
7	MW837265 *Gnathia malaysiensis*	71.3	71.2	69.5	69.9	67.8	66.9		0.322	0.329	0.296	0.333
8	MT186550 *Gnathia marleyi*	71.8	71.6	67.5	73	66.3	65.2	67.8		0.340	0.315	0.333
9	PMACA067-17 *Gnathia maxillaris*	67.2	67.3	68	67	61.3	63.4	67.1	66		0.350	0.334
10	NOISO088-15 *Gnathia oxyuraea*	71.6	71.6	72.8	70.8	69.2	69.3	70.4	68.5	65		0.298
11	AB13956 *Gnathia trimaculata*	67	66.7	68.3	66.5	66.3	74	66.2	66.3	66.2	69.8	

Column headings represent the corresponding species number in the first column of the row title. Novel sequences are indicated in bold

## Discussion

*Gnathia tridens* is here comprehensively redescribed using an integrated approach of reliable morphological techniques and successfully sequenced using both COI mtDNA and ITS2 rDNA genes. There are several discrepancies between the illustration provided by Wetzer and Brusca ([9]: Fig. 1.19) and the holotype figures given by Menzies and Barnard ([8]: Fig. 23); specifically in that the body tapers in width posteriorly (vs not tapering, mesially constricted between pereonites 3 and 4 in [8]), the dorsal surface of the cephalosome is smooth (vs granular in [8]), and the spines on the anterior margin of the cephalosome are short, apically rounded, and appear to be five (vs three spines, apically acute in [8]). These differences suggest that two species may be included under the current use of the binomen *Gnathia tridens*. Alternately, the differences may be due to drawing perspective and misinterpretation of the acute distal point of the mesioventral ridge (see Fig. 4a). Through the present study, all the defining characteristics of *G. tridens* are described in detail and illustrated using both light and scanning electron microscopy, which will eliminate further misidentification of this species.

In addition to the identification of the male, the sequences provided can also aid in the identification of female and larval specimens of *Gnathia tridens* as proven in [30], and thereafter molecular techniques can be used to link the different life stages of parasitic larvae and free-living adults of *Gnathia tridens*. Haney [10] linked several males and females of *Gnathia*, from coastal California, based on the assumption that co-occurring specimens would likely be the same species, and perceived shared morphological characters that were not verified molecularly. Further studies are needed to clarify the correct female and larval stages by combining the morphological and molecular data available. More sequences are needed to confirm the phylogenetic relationship of species within the genus *Gnathia*, and even the family Gnathiidae.

The information provided by the present study has resolved the identity of *G. tridens* and will lead to resolving the actual distribution of the species, including its supposed status as a ubiquitous Pacific coast species. The combination of a comprehensive integrated morphological description with genetic sequences will aid in clarifying future reports of this species and as well as other related species.

## Data Availability

No datasets were generated or analysed during the current study.
